# Phononic Thermal Transport along Graphene Grain Boundaries: A Hidden Vulnerability

**DOI:** 10.1002/advs.202101624

**Published:** 2021-07-21

**Authors:** Zhen Tong, Alessandro Pecchia, ChiYung Yam, Traian Dumitrică, Thomas Frauenheim

**Affiliations:** ^1^ Shenzhen JL Computational Science and Applied Research Institute Shenzhen 518131 China; ^2^ CNR‐ISMN Via Salaria km 29.300, Monterotondo Rome 00017 Italy; ^3^ Beijing Computational Science Research Center Beijing 100193 China; ^4^ Department of Mechanical Engineering University of Minnesota Minneapolis MN 55455 USA; ^5^ Bremen Center for Computational Materials Science University of Bremen Bremen 2835 Germany

**Keywords:** graphene grain boundaries, Landauer theory, molecular dynamics, phonon transport, thermal conductivity

## Abstract

While graphene grain boundaries (GBs) are well characterized experimentally, their influence on transport properties is less understood. As revealed here, phononic thermal transport is vulnerable to GBs even when they are ultra‐narrow and aligned along the temperature gradient direction. Non‐equilibrium molecular dynamics simulations uncover large reductions in the phononic thermal conductivity (*κ*_*p*_) along linear GBs comprising periodically repeating pentagon‐heptagon dislocations. Green's function calculations and spectral energy density analysis indicate that the origin of the *κ*_*p*_ reduction is hidden in the periodic GB strain field, which behaves as a reflective diffraction grating with either diffuse or specular phonon reflections, and represents a source of anharmonic phonon–phonon scattering. The non‐monotonic dependence with dislocation density of *κ*_*p*_ uncovered here is unaccounted for by the classical Klemens theory. It can help identify GB structures that can best preserve the integrity of the phononic transport.

## Graphical abstract

Contrary to conventional wisdom, phononic transport in graphene is vulnerable to grain boundaries comprising 5‐7 defects aligned along the thermal gradient. Atomistic simulations uncover a dual role of the strain field as a reflective phononic diffraction grating, showing either specular (left) or diffuse (right) phonon reflections (depending on the 5‐7 density), and as a source of anharmonic phonon–phonon scattering.

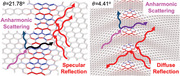

## 1 Introduction

The next generation of high‐performance electronics and sensors require materials with high thermal conductivity (*κ*_*p*_) able to spread effectively the high density of Joule heat generation along and across various thin films and substrates.^[^ ^1^, ^2^ ^]^ 2D materials including graphene are very attractive^[^ ^3^, ^4^ ^]^ for these applications as they are relatively immune to detrimental size effects on basal‐plane thermal conductivity. This is because the highly anisotropic phonon group velocity reduces the impact of scattering by the top and bottom surfaces.^[^ ^5^, ^6^ ^]^ Nevertheless, thermal transport is significantly impacted^[^ ^7^ ^]^ by defects formed during synthesis. In this respect, the widely used chemical vapor deposition (CVD)^[^ ^8^ ^]^ unavoidably produces grain boundaries (GBs). As domains nucleate randomly on substrates, their CVD growth and coalescence result in the formation of GBs.^[^ ^9^, ^10^, ^11^, ^12^ ^]^

GBs are imagined as periodic arrays of dislocations.^[^ ^13^ ^]^ In graphene, GBs are strings of pentagon‐heptagon (5‐7) edge dislocations^[^ ^10^, ^14^, ^15^, ^16^ ^]^ and their organization can gives rise to diverse GB shapes. While in general the thermal gradient can have an arbitrary orientation with respect to the GB line,^[^ ^17^, ^18^ ^]^ only transport across GBs is perceived to have a significantly impact on heat transport. Green's function (GF) calculations^[^ ^19^, ^20^ ^]^ obtained that heat transmission across GBs can be influenced by the GB structure, size, and shape. Non‐equilibrium molecular dynamics (NEMD) simulations^[^ ^21^, ^22^, ^23^ ^]^ revealed a discontinuity in the temperature (*T*) profile across GBs and that higher dislocation densities lead to lower *κ*_*p*_.

In this work, we reveal that, in fact, thermal transport is not immune to GBs oriented along the thermal gradient. By way of NEMD simulations with LAMMPS,^[^ ^24^ ^]^ we report *κ*_*p*_ reduction along GBs with various 5‐7 dislocation densities and length scales *L* between 10 to 1000 nm, in systems of up to 590 512 carbon (C) atoms treated with the optimized Tersoff potential.^[^ ^25^ ^]^ To gain a clear understanding, we further conducted GF calculations of phononic transmission and conductance, and spectral energy density (SED) calculations to quantify phonon relaxation times. The *κ*_*p*_ uncovered by these calculations displays a subtle, non‐monotonic dependence on 5‐7 defect density which is unaccounted for by the classical theory of Klemens.^[^ ^7^ ^]^

## 2 Results and Discussion

**Figure 1:**
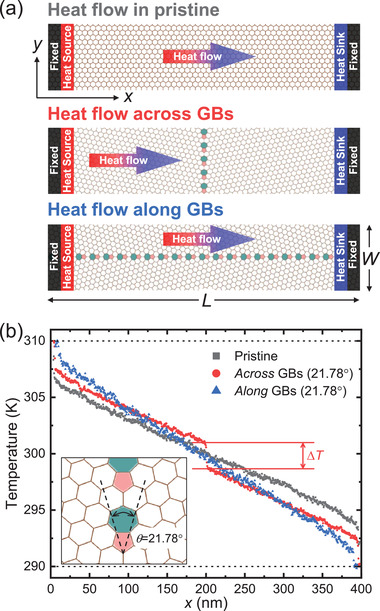
a) NEMD setup for pristine and GB graphene with *θ* = 21.78^*o*^. PBC are imposed along *y*. b) Computed *T* profiles.

The NEMD setup is presented in **Figure** **1**a. Two‐unit cells at each end were kept fixed throughout simulation and ten other neighboring unit cells were designated as “hot” and “cold” baths maintained at the temperatures *T*_*h*_ = 310 K and *T*_*c*_ = 290 K, respectively. At steady‐state, the heat flux $\dot{Q}$ was calculated as the difference of the rate of the kinetic energy extraction from the two reservoirs $\dot{Q}$ = 0.5$\langle {\dot{Q}}_{h}$‐${\dot{Q}}_{c}\rangle$, where ${\dot{Q}}_{h}$ and ${\dot{Q}}_{c}$ are the instantaneous heat currents flowing into and away from the “hot” and “cold” baths. The angular brackets indicate a statistical average taken after the steady state was reached. Graphene edges^[^ ^26^ ^]^ can significantly impact thermal transport.^[^ ^17^ ^]^ The application of periodic boundary conditions (PBC) along *y* eliminates lateral edges and allows for the simulation of the thermal transport perpendicular to a single GB line and along antiparallel GB lines (i.e, the 5‐7 defect lines run parallel to each other but with opposite directionality) separated by the lateral periodicity *W*. Therefore, differences in calculated *T* profiles, Figure 1b, can be attributed solely to GBs.

The NEMD calculations of Figure 1b at *L* = 400 nm and *W* = 15.5 nm reveal a stark difference between the *T* profiles across and along the considered GBs, which comprises aligned 5‐7 defects separated by one hexagonal ring. In agreement with Azizi et al.,^[^ ^23^ ^]^ we see a sharp temperature drop Δ*T*= 2.2 K, corresponding to a thermal resistance $\Delta T/\dot{q}$ of 0.035 Km^2^/GW across this GB. Here $\dot{q}$ is $\dot{Q}$ per cross‐sectional area (defined here based on the 0.33 nm thickness of graphene^[^ ^21^ ^]^). Along the GB line, the *T* profile is smooth and resembles the one obtained for the pristine graphene. By thermal symmetry, the GB lines oriented along the heat flow are adiabatic lines. If heat transfer was purely 1D, then *κ*_*p*_ would be hardly changed. However, the extracted $\kappa_{p}=-\dot{q}{\left(dT/dx\right)}^{-1}$, reveal a ≈50% reduction (633.2 ± 2.5 W mK^−1^ versus 1259.4 ± 7.5 W mK^−1^) demonstrating that through the two‐dimensionality of the heat transport, *κ*_*p*_ is significantly impacted even by such linear ultra‐narrow GBs.

**Figure 2:**
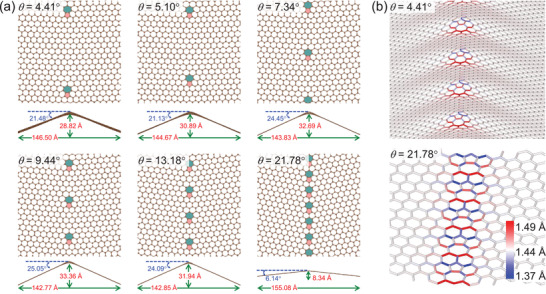
a) 6 GBs with different *θ* (top and side views). b) Bird's eye view along two GB lines. The scale shows bond lengths.

We have checked the robustness of our result (see Figure S2, Supporting Information) on a collection of symmetric tilt GB structures with similar widths *W* but different *L* and spread out linear arrangements of the 5‐7 defects, which decrease the tilt angles *θ* ^[^ ^27^ ^]^ formed by the crystallographic directions of the neighboring domains, **Figure** **2**a. It is important to note that each 5‐7 defect introduces local off‐plane elevations^[^ ^28^ ^]^ as a way of reliving the strain stored in the dislocation core. The resulting “bumpy” landscape with a rather blazed profile is visible in Figure 2b for the *θ* = 4.41° GB. The off‐plane displacements are opposite in neighboring GB lines, such that stable ripple structures are formed. As shown in the side views of Figure 2a, the ripples acquire significant amplitudes of 15±1.5Å. Only for *θ* = 21.78°, the closeness of the 5‐7 cores inhibits their off‐plane displacements reducing the ripple amplitude to only 4.17 Å. For this case, the C—C bond deformations are continuous along the GB line, Figure 2b. On the same figure, it can be also seen that the C—C bond extension and compression deformations are strongly localized around the 5‐7 cores. Overall, in all of the rippled structures of Figure 2, the C—C bond extension and compression deformations are strongly localized around the 5‐7 cores. The axial prestrain is also very small; it varies monotonically with *θ*, from −0.2% (*θ* = 4.41°) to 0.1% (*θ* = 21.78°), see Figure S1b, Supporting Information.

**Figure 3:**
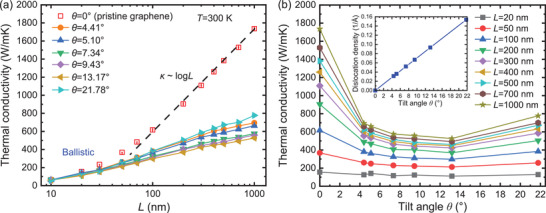
NEMD computed a) *κ*_*p*_ versus *L* (semi‐log scale) at *T* = 300 K in graphene and along GBs with different *θ*. b) *κ*_*p*_ versus *θ* at different *L*. The inset shows the 5‐7 linear density versus *θ*.

**Figure** **3**a demonstrates that the differences between *κ*_*p*_ in pristine (*θ* = 0°)^[^ ^29^, ^30^ ^]^ and along GBs with different *θ* remain significant at different *L*. In the pristine case, the initial linear increase of *κ*_*p*_, a signature of ballistic transport,^[^ ^30^ ^]^ changes (at *L*≈80 nm) into a logarithmic dependence, which is expected to hold at *L* much larger than the average phonon mean free path (≈775 nm at *T* = 300 K).^[^ ^29^, ^30^ ^]^ The unusual ≈*logL* scaling originates in the combination of reduced dimensionality of the system and the excessive population of out‐of‐plane modes.^[^ ^30^ ^]^ Along GBs, the *κ*_*p*_≈*logL* dependence is significantly attenuated such that the departure from the pristine case increases with *L*. At the largest considered *L* = 1,000 nm, the *κ*_*p*_ reduction is ≈60% or larger with respect to the pristine case. Furthermore, the *κ*_*p*_ dependence on the dislocation density is anomalous: While the dislocation density increases with *θ*, *κ*_*p*_ displays non‐monotonic variations, which become more pronounced for *L*>100 nm, Figure 3b. *κ*_*p*_ decreases from *θ* = 4.41° up to *θ* = 13.18° but presents an anomalous enhancement at *θ* = 21.78°, where the 5‐7 density is the largest.

What is the mechanism responsible for the surprising anomalous *κ*_*p*_ reduction along GBs? To answer this question we first focus on the initial ballistic regime, where we have carried out complementary GF investigations. As in NEMD, we partitioned the system into “hot” bath, device region, and “cold” bath, Figure 1a, such as the GB lines extend in all regions. We computed the ballistic transport for *θ* = 4.41°, 13.18° and 21.78° and compared them to the pristine graphene. The conductance *g* is evaluated within the Landauer approach in terms of the frequency (*ω*) dependent transmission *t*_*p*_(*ω*), as

(1) $$g=\frac{\hbar^{2}}{2\pi k_{B}T^{2}}\int \omega^{2}\frac{e^{\frac{\hbar \omega }{k_{B}T}}}{{\left(e^{\frac{\hbar \omega }{k_{B}T}}-1\right)}^{2}}t_{p}\left(\omega \right)d\omega$$

Here *k*_*B*_ and ℏ = *h*/2*π* are the Boltzmann and the reduced Planck constants, respectively. *t*_*p*_(*ω*), in turn, is computed^[^ ^31^, ^32^ ^]^ based on the dynamical matrix *D*, as *t*_*p*_ = *Tr*[*G* ^*r*^Γ_*L*_ *G* ^*a*^Γ_*R*_]. The retarded GF is given by $G^{r}\left(\omega \right)={\left[\omega^{2}-D-\Sigma_{L}^{r}-\Sigma_{R}^{r}\right]}^{-1}$ and Γ_*L*, *R*_ are the broadening functions, $\Gamma_{L/R}=i\left[\Sigma_{L/R}^{r}-\Sigma_{L/R}^{a}\right]$, for the “hot” and “cold” contacts.

**Figure 4:**
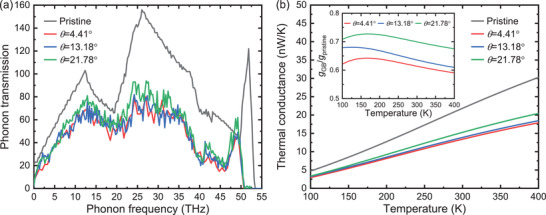
GF computed a) phonon transmissions and b) thermal conductances in pristine and along GB graphene. Inset shows GB conductances with respect to graphene.

At *L* = 10 nm considered here, transport is coherent and influenced by the elastic scattering onto the GBs. Plots of *t*_*p*_(*ω*) at different *θ* are shown in **Figure** **4**a. Consistent with NEMD, *t*_*p*_ in pristine graphene is higher than in GBs and yields a higher *g* value, Figure 4b. The ballistic nature of the conductance is reflected in the integer values of *t*_*p*_, which depend on the number of phonon states at each *ω*. Surprisingly, the *t*_*p*_(*ω*) reduction by GBs is inversely proportional to the 5‐7 density (i.e., *t*_*p*_ is largest for *θ* = 21.78° and smallest for *θ* = 4.41°). This dependence uncovers the strain field periodicity effect, which operates as a diffraction grating onto the traveling phonons. Through elastic scattering on the strain around the 5‐7 cores, reflective diffraction spectra of various orders take place. One one hand, for *θ* = 21.78°, strain is continuous along the GB line, Figure 2b. Diffraction is dominated by the zero‐order recognized by Klemens^[^ ^7^, ^33^ ^]^ and is associated with a specular reflection and larger group velocity. On the other hand, for *θ* = 4.41° GB which has lowest *g*, the 5‐7 defects are ≈3.2 nm apart, see Figure S3b, Supporting Information. Destructive interferences introduce stronger phonon localization, which is associated to diffuse reflections and manifests into important thermal resistivity contributions. These important higher diffraction orders are not considered by Klemens.^[^ ^7^, ^33^ ^]^ This situation reminds of the *κ*_*p*_ reduction along central screw dislocations located in nanowires,^[^ ^34^ ^]^ an effect also not captured by the classical theory.^[^ ^7^ ^]^ By introducing periodic nm‐scale grooves onto the nanowire surface,^[^ ^35^ ^]^ *κ*_*p*_ could be further reduced through localization of the phonons that were specularly reflected by the dislocation core.

In summary, the GF calculations obtained that the resistive contributions caused by the diffuse GB reflection scale inversely with the defect density. The *g* reductions are seen also in the inset of Figure 4b, which shows *g*_GB_/*g*_pristine_ as a function of *T*, with *g*_GB_ and *g*_pristine_ being *g* for a given GB and graphene pristine, respectively. Remarkably, the *g*_GB_/*g*_pristine_ values of Figure 4b are similar to the ones obtained earlier for transport across GBs.^[^ ^20^ ^]^ Thus, although the GBs oriented along the *T* gradient are largely overlooked, we find here that their impact on thermal transport is as substantial as the GBs oriented across the *T* gradient.

**Figure 5:**
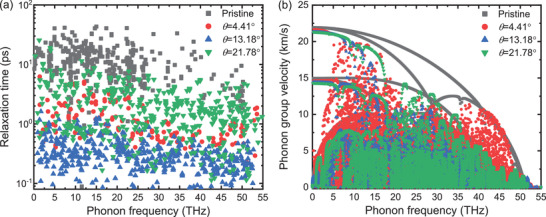
MD calculated a) phonon relaxation times at *T* = 300 K and b) phonon group velocity in pristine and GB graphene.

We now focus beyond the pure ballistic regime, where the decay of heat carrying phonons by inelastic scatterings becomes increasingly important. Recalling that in a phonon gas model, thermal conductivity is $\kappa_{p}=\sum_{\lambda }c_{\lambda }v_{\lambda }^{2}\tau_{\lambda }$, where *c*_*λ*_, *v*_*λ*_, and *τ*_*λ*_ are the specific heat capacity, phonon group velocity, and phonon relaxation time of phonon mode *λ*, respectively. **Figure** **5**a shows *τ*_*λ*_, as calculated by SED scheme^[^ ^34^, ^36^ ^]^ and room‐temperature equilibrium MD runs. When compared to pristine graphene, GBs lead to significant *τ*_*λ*_ reductions. For the “bumpy” GBs, *τ*_*λ*_ decreases with the increase in defect density. This dependence is opposite to the one for the ballistic phonon transmission delineated above, and explains the crossover in *κ*_*p*_ as transport advances into the diffusive regime. However, for *θ* = 21.78° GB, where *t*_*p*_ is the largest compared to other GBs, we also find the largest *τ*_*λ*_ in Figure 5(a). This concerted behavior explains the consistently larger *κ*_*p*_ values for *θ* = 21.78° GB with respect to the other considered GBs, Figure 3b.

The key role of *τ*_*λ*_ is further supported in Figure 5b by the lattice dynamics^[^ ^37^ ^]^ computed *v*_*λ*_, which is another key contributor to *κ*_*p*_. While for some phonon modes *v*_*λ*_ decreases in *θ* = 21.78° GB, it remains unchanged for the acoustic phonon modes, which are playing a main role in thermal conduction. Therefore, the weaker phonon scattering in *θ* = 21.78° GB (as reflected by the larger *τ*_*λ*_) and not an enhancement of *v*_*λ*_ is the mechanism behind the anomalous *κ*_*p*_ behavior. We associate the weaker anharmonic scattering presented in *θ* = 21.78° GB to the flatter landscape along the GB line. The 5‐7 off‐plane distortions at the other *θ* are enhancing anharmonic scattering as they locally couple the in‐plane and out‐of‐plane degrees of freedom,^[^ ^20^ ^]^ which are otherwise decoupled.^[^ ^38^ ^]^

## 3 Conclusion

Through the two‐dimensionality of the heat transfer, *κ*_*p*_ along linear ultra‐narrow GBs is significantly affected. The cause for the *κ*_*p*_ departure from the superdiffusive^[^ ^39^ ^]^ behavior of pristine graphene and its anomalous dependence on defect density is multifactorial, and it is hidden in the details of the GB structure. *κ*_*p*_ along *θ* = 21.78° GB is the largest, which is opposed to the expected deterioration of thermal transport at larger dislocation density.^[^ ^20^ ^]^ The *κ*_*p*_ boost is caused by a diffraction grating effect of the GB strain field periodicity, which leads to a specular scattering, and to a reduced anharmonic scattering associated to the flatter GB landscape. As the 5‐7 defects become sparse and “bumpy”, elastic scattering on GB becomes diffusive, while anharmonic scattering is enhanced. Even for the case of *θ* = 4.14° GB, where the 5‐7 defects are ≈3.2 nm apart, the *κ*_*p*_ reduction remains substantial (≈60% for *L* = 1000 nm). This explanation, enabled by state‐of‐the art atomistic‐level NEMD and GF numerical calculations with an accurate accounting for the details of the GB structure, goes beyond the simple phonon specular reflection accounted for by the classical theory of Klemens.^[^ ^7^, ^33^ ^]^ With thermal transport being vulnerable to GBs, our result can provide guidance for designing GBs that best preserve *κ*_*p*_.

## 4 Simulation Section

### Structural Relaxations

In preparation for the NEMD and GF calculations, unit cells of the GB structures treated with the optimized Tersoff potential^[^ ^25^ ^]^ and PBC along *x* and *y* directions were subjected to structural relaxations of atomic positions and lattice vectors.

### NEMD Simulation

The MD simulations used a time step of 0.5 fs and PBC along the in‐plane directions are employed in all NEMD simulations. Prior to the production runs, each configuration of graphene GB was subjected to the following protocol:^[^ ^40^, ^41^ ^]^ First, NVT simulations with Langevin thermostat^[^ ^41^ ^]^ were performed for 0.5 ns (1 × 10^6^ steps) to heat up the system to 300 K. Second, NPT simulations^[^ ^41^ ^]^ were performed for 0.5 ns to release any potential internal stress. Third, NVT simulations^[^ ^41^ ^]^ were applied for another 0.5 ns to further equilibrate the system. For the production runs, the atoms at the two sides (the black regions) are fixed to avoid the rotating of ribbons. The adjacent regions labeled with red and blue, are coupled with Langevin thermostats^[^ ^41^ ^]^ at 310 K and 290 K, respectively. The atoms in the central region are evolved in the NVE ensemble in contact with the adjacent reservoirs. We run another 10 ns (2 × 10^7^ steps) of simulation to construct the linearized temperature gradient and then record the temperature distribution and heat current data.^[^ ^41^ ^]^ The temperature gradient is obtained by the linear fitting to temperature profiles excluded the regions attached to the heat bath shown in Figure 1b. The heat flux is calculated as the heat rate per unit crossing area of GNRs, which is defined as width multiplied by the inter‐layer thickness of graphite.

### GF Calculations

Simulations used the atomistic GF method implemented in DFTB+ package.^[^ ^42^ ^]^ Based on this, a whole system with *L* = 10 nm was divided into left bath, device region, and right bath. The dynamical matrices for each subsystem were obtained within the optimized Tersoff potential^[^ ^25^ ^]^ description of the interatomic interactions with a finite difference scheme of the atomic forces, not accounting for finite temperature phonon softening effects owing to anharmonicity.

### SED Calculations

The SED analysis^[^ ^43^ ^]^ is employed to obtain the phonon relaxation time. Based on this, the equilibrium MD was carried out to compute the velocity of atoms and then obtained the peaks of SED spectrum for different phonon branches, see Figure S4, Supporting Information. The Lorentzian function fitting technique was employed to determine the phonon relaxation time. SED calculations are performed using an in‐house code.

### Statistical Analysis

The results in Figure 3 are obtained by averaging results obtained from five individual NEMD trajectories of each GB system. The length (width) of each system *L* (*W*) is given in Figure 3 (Figure 2a). Results are represented as means ± SD.

## Conflict of Interest

The authors declare no conflict of interest.

## Supporting information

Supporting Information

## Data Availability

The data that support the findings of this study are available from the corresponding authors upon reasonable request.
