# Supplementary material for: Phononic Thermal Transport along Graphene Grain Boundaries: A Hidden Vulnerability
Source: Adv Sci (Weinh). 2021 Jul 21;8(18):2101624. doi: 10.1002/advs.202101624 (PMC8456227; doi:10.1002/advs.202101624)
Supplement: Supplementary file 1 — Supporting Information [file ADVS-8-2101624-s001.pdf]

© 2021 Wiley-VCH GmbH

ADVANCED SCIENCE
Open Access

Supporting Information

for *Adv. Sci.*, DOI: 10.1002/advs.202101624

Phononic thermal transport along graphene grain boundaries: A hidden vulnerability

Zhen Tong Alessandro Pecchia ChiYung Yam Traian Dumitrica, and Thomas Frauenheim*

Supplementary Material

Phononic thermal transport along graphene grain boundaries: A hidden vulnerability

Zhen Tong,[1] Alessandro Pecchia,[2] ChiYung Yam,[3,4]
Traian Dumitrică,[5,*] and Thomas Frauenheim[4,3,6,†]

[1]Shenzhen JL Computational Science and Applied Research Institute, Shenzhen 518113, China.
[2]CNR-ISMN, Via Salaria km 29.300, Monterotondo 00017, Rome, Italy
[3]Shenzhen JL Computational Science and Applied Research Institute, Shenzhen 518110, China.
[4]Beijing Computational Science Research Center, Beijing 100193, China
[5]Department of Chemical Engineering and Materials Science, Scientific Computation Program, and Department of Mechanical Engineering, University of Minnesota, Minnesota 55455, United States
[6]Bremen Center for Computational Materials Science, University of Bremen, Bremen 2835, Germany

S1. Translation vector parameters for GBs

Generally, the atomic structure of grain boundaries (GBs) in graphene can be defined by θ_R and θ_L, the rotated angles between the corresponding crystallographic directions in the two domains (right and left) and the normal of the boundary line [1, 2] as shown in Fig. **S1**(a). As employed in Ref. [1, 2], the translation vectors (n_R, m_R) and (n_L, m_L) of right and left domains along the defect line are defined to construct the periodically arranged defects in graphene. Therefore, the tilt angle for a certain GB graphene structure can be obtained by $\theta = 60^\circ - (\theta_\mathrm{R} + \theta_\mathrm{L}) = \arccos(\sqrt{3}(n_\mathrm{R} + m_\mathrm{R})/2\sqrt{n_\mathrm{R}^2 + m_\mathrm{R}^2 + n_\mathrm{R}m_\mathrm{R}}) + \arccos(\sqrt{3}(n_\mathrm{L} + m_\mathrm{L})/2\sqrt{n_\mathrm{L}^2 + m_\mathrm{L}^2 + n_\mathrm{L}m_\mathrm{L}})$. The parameters for the symmetric $(n_\mathrm{R}, m_\mathrm{R})|(n_\mathrm{L}, m_\mathrm{L})$ GB in this work are provided in Table **S1**.

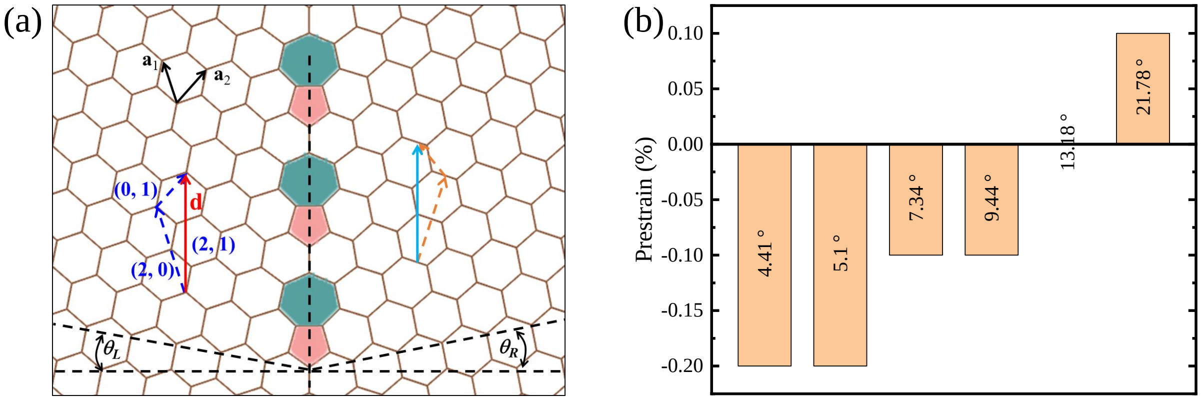

Fig. S1: (a) An example of a tilt GB in graphene separating two crystalline domains rotated by $\theta = 60^\circ - (\theta_\mathrm{R} + \theta_\mathrm{L}) = 60^\circ\text{-}(19.11^\circ\text{+}19.11^\circ)\text{=}21.78^\circ$ with respect to each other. According to Ref. [1], the repeat vector $\mathbf{d} = n\mathbf{a}_1 + m\mathbf{a}_2$ of the grain-boundary structure is defined by the matching vectors (2, 1) and (2, 1) in the left and right domains, respectively. A possible atomic structure of the interface region involves one elementary dislocation dipole (pentagon-heptagon pairs) per repeat cell. (b) Prestrain of the graphene nanoribbons with GBs.

Table S1: Parameters of translation vectors for GBs constructed in this work.

Tilt angle (θ)	4.41°	5.10°	7.34°	9.44°	13.18°	21.78°
$(n_\mathrm{R}, m_\mathrm{R})\|(n_\mathrm{L}, m_\mathrm{L})$	(8,7)|(8,7)	(7,6)|(7,6)	(5,4)|(5,4)	(4,3)|(4,3)	(3,2)|(3,2)	(2,1)|(2,1)

S2. Validation of NEMD calculations

In order to validate the optimized Tersoff potential [3] used in the MD calculations, we calculate the phonon dispersion of pristine graphene and compare it with reported data as shown in Fig. **S2**(a). The experimental data are obtained from graphite, which are taken from Refs. [4] (yellow diamond dot) and [5] (green triangle dot). Our calculations agree well with experimental data, indicating the feasibility of the optimized Tersoff potential [3] for describing the interactions between carbon atoms in graphene. Furthermore, we calculated the Kapitza resistance [6] of GNRs at the interface between grain domains for heat flux across GB and compared the results with reported data [7, 8] as shown in Fig. **S2**(b). Our predictions agree well with reported data, indicating the reliability of the NEMD calculations implemented in this work.

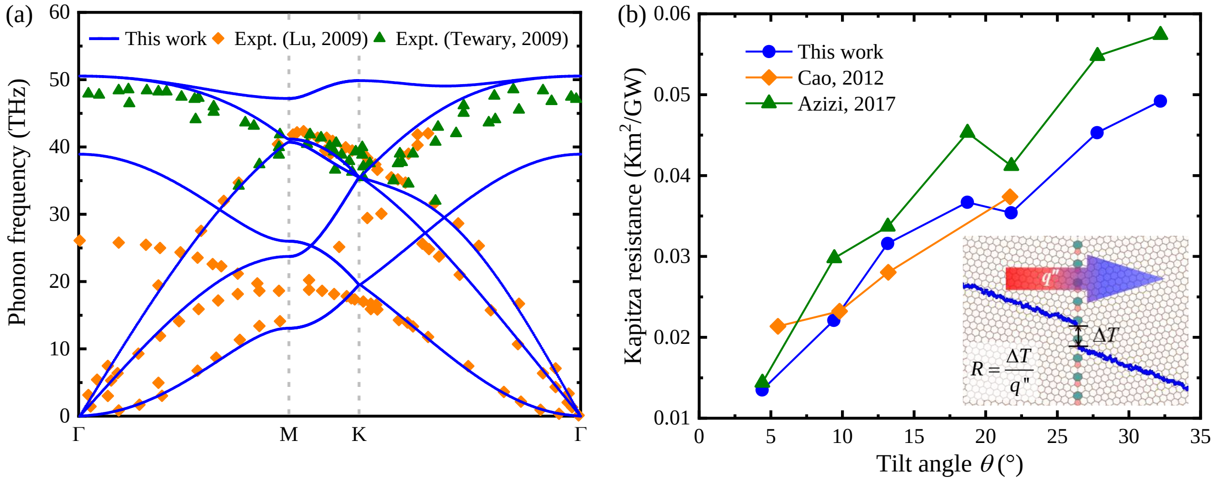

Fig. S2: (a) Phonon dispersion from lattice dynamics. The experimental data are taken from Refs. [4] (yellow diamond dot) and [5] (green triangle dot). (b) Thermal conductivity of GNRs for heat flow perpendicularly to GBs. The reported data are referred to Refs. [7] (yellow diamond dot) and [8] (green triangle dot)

S3. Phonon transmission from Green's function

We calculated the transmissions of the curved structures is essentially the same to those of perfectly flat graphene with the same GB defects, as seen in Fig. **S3**.

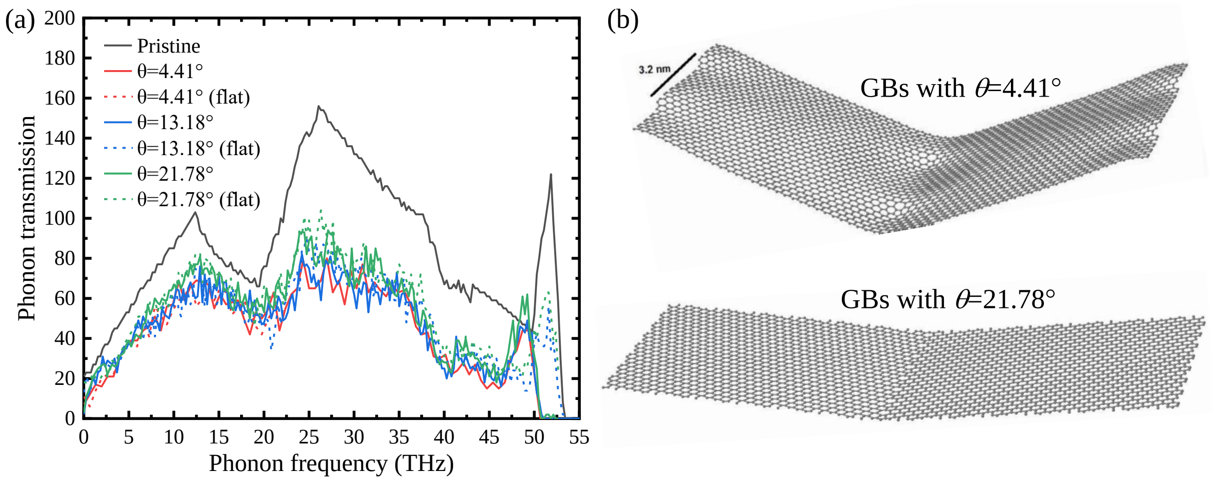

Fig. S3: (a) Calculated phonon transmission from Green's function of pristine graphene ($\theta = 0°$). and of 4.41°-GB GNRs, 13.18°-GB GNRs, and 21.78°-GB GNRs for bent and flat structures. (b) Structure features of localized dislocations for the case of θ=4.41° and θ=21.78°.

S4. Computational details of SED

The phonon relaxation time can be obtained by performing the spectral energy density (SED) analysis [9–12] based on equilibrium MD simulations. The SED is expressed as

$$\Phi(\mathbf{k},\omega) = \sum_{\nu}^{3n} \Phi_\nu(\mathbf{k},\omega) = \frac{1}{4\pi\tau_0} \sum_{\alpha}^{3} \sum_{b}^{n} \frac{m_b}{N_c} \left| \sum_{l}^{N_c} \int_0^{t_0} \dot{u}_\alpha^{l,b}(t) \exp(i\mathbf{k}\cdot\mathbf{r}_0^l - i\omega t) dt \right|^2, \tag{1}$$

where $\mathbf{k}$ is the wave vector, ω is the frequency, ν is the phonon branch, τ_0 is the integration time constant. Index α represents x, y, and z directions, b is the index of basis atoms, l is the index of unit cells, N_c is the total number of unit cells, m_b is the atomic mass of basis atom b. $u_\alpha^{l,b}$ is the αth component of the displacement of the bth basis atom in the lth unit cell. The shape of $\Phi_\nu(\mathbf{k},\omega)$ for each phonon branch can be taken as a Lorentzian function

$$\Phi_\nu(\mathbf{k},\omega) = \frac{C_{\mathbf{k},\nu}}{(\omega - \omega_{\mathbf{k},\nu}^A)^2 + \Gamma_{\mathbf{k},\nu}^2}. \tag{2}$$

where $C_{\mathbf{k},\nu}$ is a constant related to the Lorentzian peak magnitude, $\omega_{\mathbf{k},\nu}^A$ is the frequency at the peak center, and $\Gamma_{\mathbf{k},\nu}$ is the half-width at half-maximum. The phonon relaxation time is then obtained by $\tau_{\mathbf{k},\nu} = 1/2\Gamma_{\mathbf{k},\nu}$. Submitting the velocity from MD simulations to Eq. (1) to obtain the SED peaks for different phonon branches, which results in the determination of $\omega_{\mathbf{k},\nu}^A$ and $\Gamma_{\mathbf{k},\nu}$ by employing the Lorentzian function fitting. All the SED calculations are performed using an in-house code.

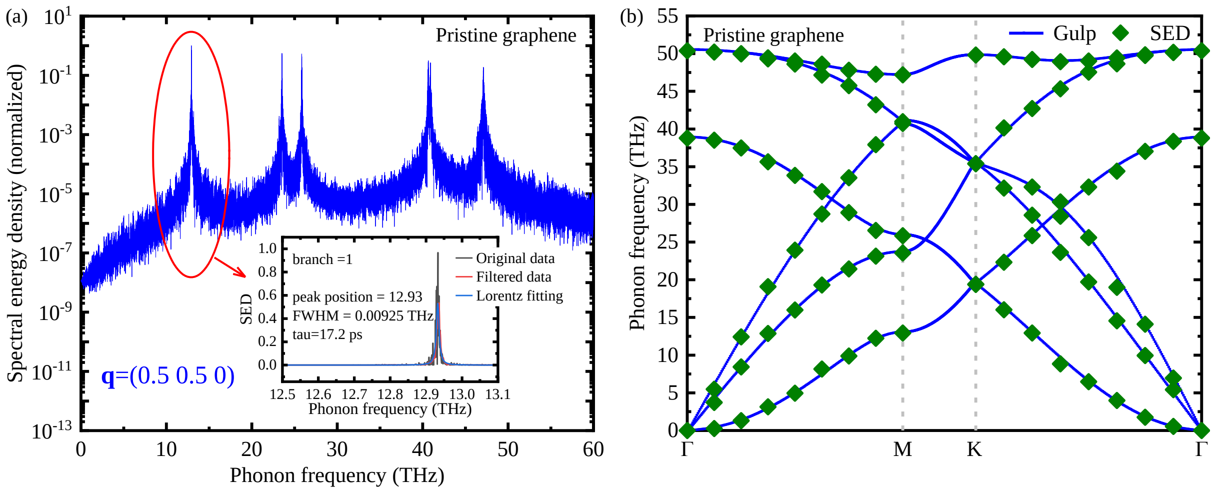

Fig. S4: (a) Calculated spectral energy density at 300 K of pristine graphene at M point [$\mathbf{q} = (0.5, 0.5, 0)$] in the BZ, the inset is the Lorentzian function fitting of the first branch at M point in graphene. (b) Phonon frequency in the BZ in pristine graphene, in which the lattice dynamics calculations using GULP [13] (blue solid line) and the SED fitted data (olive diamond dot).

In order to obtain the phonon relaxation time, the Lorentzian function fitting of the SED is employed. In this work, the SED of graphene with tilt angles $\theta = 0^\circ$ (pristine), $\theta = 4.41^\circ$, and $\theta = 21.78^\circ$ are calculated for comparison. The simulation domains for those structures are 1440 conventional cells (2 atoms in unitcell) for pristine graphene (2880 atoms), 62 conventional cells along the GB (268 atoms in unitcell) for $\theta = 4.41^\circ$ GB (16616 atoms), 120 conventional cells along the GB (100 atoms in unitcell) for $\theta = 13.18^\circ$ GB (12000 atoms), and 306 conventional cells along the GB (52 atoms in unitcell) for $\theta = 21.78^\circ$ GB (15912 atoms). Nine $\mathbf{q}$ points with the reduced wave vector being $\mathbf{q}^* = (\eta/16, \eta/16, 0)$ and $\mathbf{q}^* = (\eta/24, \eta/12, 0)$ are resolved for $\Gamma \to$ M and K $\to \Gamma$ in the Brillouin zone (BZ) for pristine graphene ($\theta = 0$), where η is an integer from 0 to 8. For $\theta = 4.41^\circ$, $\theta = 13.18^\circ$ and $\theta = 21.78^\circ$ GBs, five $\mathbf{q}$ points with $\mathbf{q}^* = (\zeta/8, 0, 0)$ are resolved along the GBs direction, where ζ is an integer from 0 to 4. As an example, the calculated spectral energy density at 300 K of pristine graphene at M point [$\mathbf{q} = (0.5, 0.5, 0)$] in the BZ are shown in Fig. S4(a), in which the Lorentzian function fitting of the first branch is provided in the inset. In addition, the SED fitted phonon frequencies in the BZ of pristine graphene are compared with the phonon frequency calculated from lattice dynamics theory employed in GULP [13] as shown in Fig. S4(b) and they are in well agreement, indicating the accuracy and reliability of the SED calculations.

* Electronic address: dtraian@me.umn.edu
† Electronic address: thomas.frauenheim@bccms.uni-bremen.de
[1] O. V. Yazyev and S. G. Louie, *Topological defects in graphene: Dislocations and grain boundaries*, Physical Review B **81**, 195420 (2010).
[2] O. V. Yazyev and S. G. Louie, *Electronic transport in polycrystalline graphene*, Nature Materials **9**, 806 (2010).
[3] L. Lindsay and D. A. Broido, *Optimized Tersoff and Brenner empirical potential parameters for lattice dynamics and phonon thermal transport in carbon nanotubes and graphene*, Physical Review B **81**, 205441 (2010).
[4] Q. Lu, M. Arroyo, and R. Huang, *Elastic bending modulus of monolayer graphene*, Journal of Physics D: Applied Physics **42**, 102002 (2009).
[5] V. K. Tewary and B. Yang, *Parametric interatomic potential for graphene*, Physical Review B **79**, 075442 (2009).
[6] G. L. Pollack, *Kapitza Resistance*, Reviews of Modern Physics **41**, 48 (1969).
[7] A. Cao and J. Qu, *Kapitza conductance of symmetric tilt grain boundaries in graphene*, Journal of Applied Physics **111**, 053529 (2012).
[8] K. Azizi, P. Hirvonen, Z. Fan, A. Harju, K. R. Elder, T. Ala-Nissila, and S. M. V. Allaei, *Kapitza thermal resistance across individual grain boundaries in graphene*, Carbon **125**, 384 (2017).
[9] J. A. Thomas, J. E. Turney, R. M. Iutzi, C. H. Amon, and A. J. H. McGaughey, *Predicting phonon dispersion relations and lifetimes from the spectral energy density*, Physical Review B **81**, 081411(R) (2010).
[10] B. Qiu, H. Bao, G. Zhang, Y. Wu, and X. Ruan, *Molecular dynamics simulations of lattice thermal conductivity and spectral phonon mean free path of PbTe: Bulk and nanostructures*, Computational Materials Science **53**, 278 (2012).
[11] Y. Ni, S. Xiong, S. Volz, and T. Dumitrică, *Thermal Transport Along the Dislocation Line in Silicon Carbide*, Physical Review Letters **113**, 124301 (2014).
[12] T. Feng, B. Qiu, and X. Ruan, *Anharmonicity and necessity of phonon eigenvectors in the phonon normal mode analysis*, Journal of Applied Physics **117**, 195102 (2015).
[13] J. D. Gale and A. L. Rohl, *The General Utility Lattice Program (GULP)*, Molecular Simulation **29**, 291 (2003).
